# P-357. The Global REGAL cohort: A REtrospective real-world study of the effectiveness and tolerability of the antiretroviral treatment reGimens DTG/3TC compAred to BIC/FTC/TAF in older persons Living with HIV

**DOI:** 10.1093/ofid/ofaf695.575

**Published:** 2026-01-11

**Authors:** Jeremy Fraysse, Jennifer Kuretski, Emilio Letang, Maria Jesus Perez Elías, Gustavo Verdier, Qingxia Zhao, Eva Fernvik, Jun Yong Choi, Richard Grove, Chien-Yu Cheng, Cynthia Firnhaber, Arnaud Desclaux, Axel Baumgarten, Eleanora Zonta, Mark Lynam, Bryn Jones, Julie Priest

**Affiliations:** ViiV Healthcare, Durham, NC; Midway Specialty Care Center, West Palm Beach, Florida; ViiV Healthcare, Madrid, Spain, Madrid, Madrid, Spain; Hospital Universitario Ramón y Cajal, Madrid, Madrid, Spain; ViiV Healthcare, Montréal, QC, Canada, Pointe-Claire, Quebec, Canada; Zhengzhou Municipal Sixth People’s Hospital and Infectious Disease Hospital of Henan Province, Zhengzhou, Henan, China; ViiV Healthcare, Durham, NC; Yonsei University College of Medicine, Seoul, Seoul-t'ukpyolsi, Republic of Korea; GSK, London, England, United Kingdom; Taoyuan General Hospital, Taoyuan, Taoyuan, Taiwan; University of Colorado Anschutz Medical Center, Denvier, Colorado; CHU Bordeaux, Bordeaux, Aquitaine, France; Zentrum für Infektiologie Berlin Prenzlauer Berg, Berlin, Berlin, Germany; IQVIA, Barcelona, Catalonia, Spain; IQVIA, Barcelona, Catalonia, Spain; ViiV Healthcare, Durham, NC; ViiV Healthcare, Durham, NC

## Abstract

**Background:**

Older persons living with human immunodeficiency virus (PLHIV) have more age-related comorbidities than the general population of PLHIV and greater potential for polypharmacy and drug-drug interactions with antiretroviral treatment (ART). Data comparing the real-world effectiveness of the two-drug dolutegravir/lamivudine (DTG/3TC) and three-drug bictegravir/emtricitabine/tenofovir alafenamide (BIC/FTC/TAF) is limited in older PLHIV.

**Figure:**
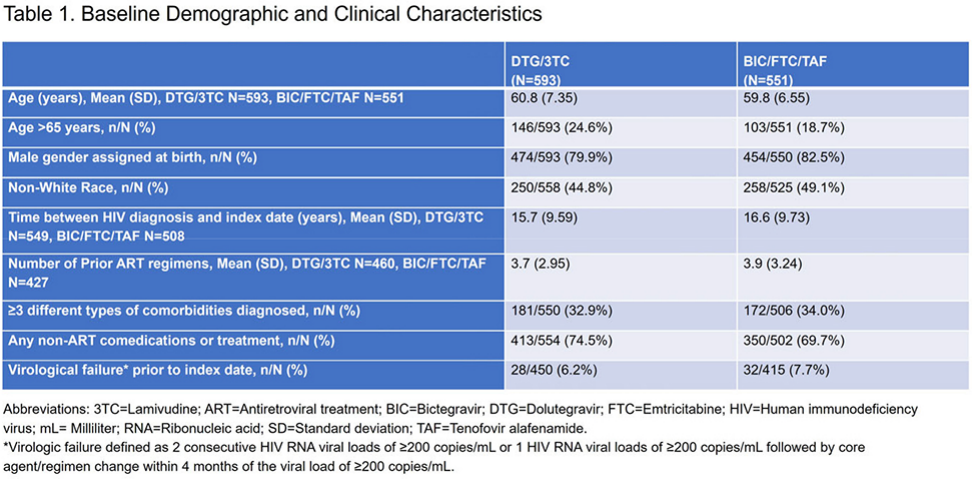

**Figure:**
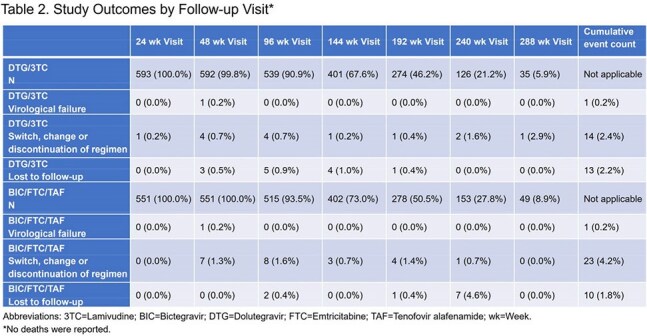

**Methods:**

To assess the real-world effectiveness of switching to DTG/3TC versus BIC/FTC/TAF in older PLHIV, we conducted a retrospective chart review among people aged ≥50 years on ART who were virologically suppressed with ≥24 weeks of follow-up across 7 countries. Index date was defined as DTG/3TC or BIC/FTC/TAF initiation date.

**Results:**

Among 1144 PLHIV (326 United States, 221 China, 189 Spain, 140 Germany, 113 France, 100 Korea, 55 Taiwan), 593 were on DTG/3TC and 551 were on BIC/FTC/TAF. Demographic and clinical characteristics were similar between groups at index date (Table 1). Among PLHIV on DTG/3TC and BIC/FTC/TAF, 24.6% and 18.7%, were >65 years and 74.5% and 69.7% reported a non-ART comedication, respectively. Prior virological failure was reported in 6.2% of DTG/3TC and 7.7% of PLHIV on BIC/FTC/TAF. Total follow-up was 1463.3 and 1481.9 person-years among DTG/3TC and BIC/FTC/TAF groups, respectively. Approximately 25% of PLHIV had 240 weeks of follow-up. Virological failure was reported for 1 person in each group. The incidence rate of virological failure was 0.07 (95% confidence interval: 0.00-0.14) per 100 person-years for both groups. No resistance was reported.

**Conclusion:**

In older, virologically suppressed PLHIV with age-related comorbidities and comedications, switching to either two-drug DTG/3TC or three-drug BIC/FTC/TAF maintained long-term viral suppression without resistance.

**Disclosures:**

Jeremy Fraysse, MS, GSK: Stocks/Bonds (Public Company)|ViiV Healthcare: Stocks/Bonds (Private Company) Jennifer Kuretski, DNP, APRN, NP-C, AAHIVS, Gilead Sciences: Speaker|ViiV: Speaker Emilio Letang, MD, MPH, PhD, GSK: Stocks/Bonds (Public Company)|ViiV Healthcare: Employee Maria Jesus Perez Elías, MD, PhD, Gilead: Advisor/Consultant|Gilead: Grant/Research Support|Gilead: Honoraria|Janssen Cilag: Advisor/Consultant|Janssen Cilag: Grant/Research Support|Janssen Cilag: Honoraria|ViiV healthcare: Advisor/Consultant|ViiV healthcare: Grant/Research Support|ViiV healthcare: Honoraria Gustavo Verdier, BSc, BPharm, MBA, ViiV Healthcare: Employee Eva Fernvik, PhD, GSK: Stocks/Bonds (Public Company)|ViiV Healthcare: Stocks/Bonds (Private Company) Richard Grove, BSc, MSc, GSK: Stocks/Bonds (Public Company) Cynthia Firnhaber, MD, MS, DTM&H, MERCK: Advisor/Consultant|MERCK: Grant/Research Support Axel Baumgarten, MD, Abbvie: Board Member|Astra Zeneca: Board Member|Gilead: Advisor/Consultant|Gilead: Board Member|Gilead: Honoraria|GSK/ViiV Healthcare: Board Member|Janssen: Board Member|MSD: Board Member|Pfizer: Board Member Bryn Jones, MBChB, GSK: Stocks/Bonds (Public Company)|ViiV Healthcare: Employee Julie Priest, MSPH, GSK: Stocks/Bonds (Public Company)|ViiV Healthcare: Employee

